# Estimates of the global workforce required for providing assistive technology: a modeling study

**DOI:** 10.3389/fresc.2025.1617624

**Published:** 2025-07-08

**Authors:** Johanna Rosberg Petersson, Malin Tistad, Sébastien Muller, Irene Calvo, Johan Borg

**Affiliations:** ^1^ School of Health and Welfare, Dalarna University, Falun, Sweden; ^2^ Department of Neurobiology, Care Sciences and Society, Karolinska Institutet, Huddinge, Sweden; ^3^ Health, SINTEF Digital, Trondheim, Norway; ^4^ Access to Assistive Technology (ATA), Medicines and Health Products, World Health Organization, Geneva, Switzerland

**Keywords:** assistive products, assistive technology, need, access, workforce

## Abstract

**Introduction:**

Despite being a fundamental human right, access to assistive products varies between 3% and 90% across countries. Ensuring adequate and trained human resources is a prerequisite for improving access to assistive products. To support workforce planning and development, this study estimated the global workforce required for assistive technology provision to achieve a high level of access.

**Method:**

This modeling study used estimates of the primary workforce for assistive technology provision and assistive product needs in a country with a high level of access and global assistive product needs, to predict the global workforce required to provide assistive technology in five product domains: cognition and communication, hearing, mobility and self-care, orthotics and prosthetics, and vision. The assistive product need estimates were based on self-reported data from WHO Rapid Assistive Technology Assessment surveys in 28 countries.

**Results:**

A total workforce for assistive technology provision of 4.4 (95% CI: 3.0–6.8) million full-time equivalents (FTE) would be required globally to achieve a high level of access to assistive products. Excluding the administrative workforce, this includes a workforce of 3.4 (2.3–5.4) million FTE, composed of 1.7 (1.3–2.2) million FTE providing mobility and self-care products, 0.9 (0.5–1.7) million FTE providing orthoses and prostheses, 0.5 (0.2–1.0) million FTE providing vision products, 0.3 (0.2–0.4) million FTE providing hearing products, and 0.05 (0.04–0.06) million FTE providing cognition and communication products.

**Conclusion:**

Likely a conservative estimate of the required workforce size, this provides a cautious foundation for informing strategies to develop a workforce capable of meeting global assistive product needs and improving access.

## 1 Introduction

More than 2.5 billion people globally need assistive products today. This need is projected to increase to 3.5 billion in 2050 due to aging and non-communicable diseases (1). Assistive products are products external to the body whose primary purpose is to maintain or improve an individual's functioning and independence and thereby promote well-being. They can also prevent impairments and secondary health conditions (2). Assistive products can facilitate individuals' inclusion and participation in society, and their access to health, education, work, and other important areas of life by providing support in various human functional domains, such as cognition, communication, self-care, hearing, mobility, and vision (1, 3). Using assistive products also benefits families and society both financially and health-wise by, for example, paid work and reduced stress (4, 5). Each unit invested in increasing access to assistive products is estimated to give a nine-times economic return (5).

In many countries, individuals' access to assistive products, defined as the ratio of prevalence of self-reported met need to prevalence of self-reported need, is low. Based on surveys in 29 countries, access to assistive products ranged between 3% and 90% when ordinary spectacles were included, and 2%–84% when ordinary spectacles were excluded (1). Barriers to accessing assistive products include, for example, lack of awareness and limited availability of information, high costs, limited physical and geographical access, inadequate products, limited funding, and workforce shortage (1, 6, 7).

Assistive technology includes assistive products and systems and services for their provision (1). In the WHO GATE 5P framework of assistive technology, the workforce is an integral part of the component personnel, interlinking with the other four components: person, provision, product, and policy (8). These five components are all crucial to achieving universal access to assistive technology, where personnel, provision, and policies can be conceptualized as a bridge between a person in need and the assistive product (9). Despite the importance of having an adequate workforce, an investigation of the system preparedness for assistive technology provision found that only 7 of 70 participating countries reported having an adequate and trained workforce for all levels of services, and for people with difficulties in all functional domains (1).

The workforce involved in assistive technology provision includes a diverse range of occupations, such as medical doctors, nurses, occupational therapists, social workers, audiologists, speech and language therapists, orthotists, prosthetists, physiotherapists, and opticians (10, 11). They may work with persons requiring assistive products to address needs in one or more functional domains (11). In addition, other occupations, such as technicians, administrators, and other service personnel, support their work (12).

Shortages in the workforce for assistive technology provision occur in countries irrespective of their income level (12, 13). There might also be differences in the geographical distribution of the workforce within a country, with a lower density in rural areas and areas with lower socioeconomic status (13, 14). Having an adequate and trained workforce is positively correlated with access to assistive products (9). Therefore, in its resolution on improving access to assistive technology, the 71st World Health Assembly urges countries to ensure the availability of adequate and trained human resources (15). However, to our knowledge, no attempt has been made to estimate the size of such a workforce. In orthotics and prosthetics, it has been suggested that an average country requires 5–10 orthotists and prosthetists per million people, along with twice as many orthotic and prosthetic technicians and support staff to meet the demand for orthoses and prostheses (16).

Effective planning and development of a global workforce for assistive technology provision necessitate understanding the required scale to ensure adequate access. Accordingly, this study aimed to estimate the global assistive technology provision workforce required to achieve a high level of access to assistive products.

## 2 Methods

This modeling study utilized cross-sectional data on the assistive technology provision workforce from three regions within a country with a high level of access to assistive products. It also incorporated estimates of assistive product needs, both within that country and globally, disaggregated across five product domains, largely corresponding to domains of human functioning.

### 2.1 Context

Sweden was selected for the collection of workforce data for three reasons. It was the country with the highest level of access to assistive products (84% when excluding ordinary spectacles) among the countries included in the WHO UNICEF Global Report on Assistive Technology (1). It has reported having adequate and trained human resources at all levels of service delivery and for all functional domains (9). Moreover, the workforce density of medical doctors and nurses in Sweden is close to the mean workforce density in countries with a Universal Health Coverage Service Coverage Index in Sweden or higher (17, 18), indicating that the workforce productivity in the health sector is on par with countries with similar achievements in terms of service coverage. These considerations suggest that the size of the workforce for assistive technology provision in Sweden is relevant to this study. To provide context for interpreting the workforce data, a brief overview of Sweden's assistive technology provision system follows.

Regulated by law, the responsibility for providing assistive technologyis shared between 21 healthcare regions and 290 municipalities with their respective self-governing local authorities (19, 20). Most assistive products are provided based on the Health and Medical Services Act, but assistive products can also be provided based on the Social Care Act and legislation related to housing, education, and work (19, 21).

This study focuses on the primary segment of the workforce responsible for providing assistive technology under the Health and Medical Services Act. They typically operate within one of five product domains that underpin the structure of the assistive technology provision system: cognition and communication, hearing, mobility and self-care, orthotics and prosthetics, and vision. Occupations prescribing assistive products are usually health professionals. They assess the need for assistive products and are responsible for the prescription process, including assessment, testing, adaptation of the assistive product, information, training, follow-up, and evaluation (19). If the prescriber does not have adequate skills, a counselor can be hired or a referral made to another health professional with the appropriate skills (19). However, other personnel, such as technicians, are involved during the prescription process.

### 2.2 Data

#### 2.2.1 Data on the workforce in Sweden

To estimate the size of the assistive technology workforce in Sweden, data on occupational categories were collected from assistive technology managers in three healthcare regions. These regions were selected through convenience sampling to represent varying sizes and urban-rural characteristics. The reported workforce data were divided into a clinical and non-clinical workforce, and an administrative workforce. The occupations are presented below according to the sub-major groups of the International Standard Classification of Occupations (ISCO-08), followed by occupations in brackets (22).

*Clinical and non-clinical workforce*:

• Health professionals (audiologists, opticians, physiotherapists, dietitians, occupational therapists, speech and language therapists)
• Health associate professionals (orthotists, prosthetists, orthotic technicians, prosthetic technicians, audiometric equipment technicians, assistant nurses)
• Legal, social, and cultural professionals (psychologists, social workers, sign language interpreters)
• Teaching professionals (educators)
• Science and engineering professionals (engineers, technicians)
• Garment and other craft and related trades workers (repairers, orthopedic shoemakers, seamstresses, technicians)
• Laborers in mining, construction, manufacturing, and transport (warehouse porters)
• Drivers and mobile plant operators (drivers)
• Counselors, i.e., health professionals and health associate professionals in the Swedish centers for assistive technology provision (an occupation not classified in ISCO-08)

*Administrative workforce*:

• Administrative and commercial managers (managers)
• Production and specialized services managers (managers, administrators)
• Business and administration associate professionals (secretaries, purchasers, assistants)
• Customer service clerks (receptionists)

Two of the healthcare regions also provided workforce data on prescribers, i.e., personnel prescribing assistive products for cognition, communication, mobility, or self-care as part of their duties, but who do not work in the centers responsible for assistive technology provision.

To enable triangulation of the national workforce estimates, personnel statistics were also obtained from the Swedish Association of Local Authorities and Regions (special data retrieval, unpublished data). According to their records, approximately 6,400 individuals worked with assistive technology provision in the healthcare regions in Sweden, corresponding to 5,470 full-time equivalents (FTE). However, this may be an underestimation, as some staff may have been classified under other workforce categories. Moreover, the Swedish Association of Local Authorities and Regions lacked data on the assistive technology workforce within municipalities, suggesting that the total workforce in Sweden was likely higher. Regarding orthotists and prosthetists, workforce density data from the Swedish National Board of Health and Welfare indicated 5 FTE per 100,000 population (23).

The workforce providing ordinary spectacles was excluded as they are part of another provisioning system. The workforces providing or prescribing assistive products for work and education were also excluded, as these products are provided by the Swedish Public Employment Service, the Social Insurance System, or the education system, which is separate from the healthcare system.

#### 2.2.2 Data on assistive product needs

This study used data from representative WHO Rapid Assistive Technology Assessment (rATA) surveys conducted in 28 countries (Azerbaijan, Bhutan, Burkina Faso, China, Djibouti, Dominican Republic, Georgia, Guatemala, India, Indonesia, Iran, Iraq, Italy, Jordan, Kenya, Liberia, Malawi, Maldives, Mongolia, Myanmar, Nepal, Poland, Rwanda, Senegal, Sweden, Tajikistan, Togo, and Ukraine) during 2020 and 2021 involving 260,924 participants (1). Among them, 51.4% were females, 30.4% were 0–17 years old, 55.0% were 18–59 years old, and 14.7% were 60 years and older. The method for collecting data on access to assistive products, excluding spectacles, is described in the Global Report on Assistive Technology and by Zhang et al. (1, 24).

rATA is a population-based household survey for mapping self-reported need for, demand for, supply of, barriers to, and user satisfaction with assistive products (25). The rATA instrument has been examined for face and construct validity and deemed appropriate for use in different contexts (24). One key indicator of the rATA survey is the *prevalence of need*, which is the sum of the *prevalence of met need* and the *prevalence of unmet need*. The prevalence of met need is the proportion of a population using assistive products that do not need new or additional assistive products, while the prevalence of unmet need is the proportion of a population that needs new or additional assistive products, regardless of using an assistive product.

The functional domains used to categorize assistive products in rATA were rearranged to align with the organization of the centers for assistive technology provision in Sweden. The rATA domains *hearing* and *vision* (without ordinary spectacles) were used as product domains without any changes. The rATA domains cognition and communication were merged into the product domain *cognition and communication*, while mobility was divided into mobility excluding orthoses and prostheses, and the product domain *orthoses and prostheses*. Mobility, excluding orthoses and prostheses, was then merged with the rATA domain self-care, resulting in the product domain *mobility and self-care*.

#### 2.2.3 Population data

In July 2023, the total population in Sweden was 10,551,707 persons with a median age of 41.0 years. Of the total population, 2,176,224 persons were between 0 and 17 years old. In the three regions for which workforce data from centers providing assistive technology were collected, the combined population was 936,923 persons, corresponding to approximately 8.9% of the total population in Sweden (26, 27). The median ages of the populations in the three regions varied between 41 and 45 years (27).

The global population in July 2023 was 8,091,735,000 persons (28).

### 2.3 Data analysis

The data analysis was conducted in three steps and disaggregated across the five product domains. First, the workforce at the centers for assistive technology provision in Sweden was estimated using data from the three healthcare regions. Second, the assistive product needs were estimated for both Sweden and the world. Third, the size of the required provision workforce to meet the global need for assistive products was calculated.

#### 2.3.1 Workforce in Sweden

The estimated workforce in Sweden (*W_S_*) was calculated using workforce data from the three healthcare regions (*W_R_*) and the ratio between the national population (*P_S_*) and the population in the three healthcare regions (*P_R_*). Thus, the total workforce and the clinical and non-clinical workforce, respectively, were calculated for each product domain using the formula:

$$W_{S}=W_{R}\frac{P_{S}}{P_{R}}.$$

#### 2.3.2 Needs for assistive products in Sweden and globally

In 26 of the 28 surveyed countries, the number of people in need of assistive products was calculated based on the prevalence of need for assistive products and the size of the population. As the participants included in the Swedish and Dominican Republic rATA surveys were 18 years and older, the need estimates in these countries were the sum of the prevalence of need multiplied by the size of the population aged 18 and a smaller prevalence of need multiplied by the size of the population below 18 years of age. For the Dominican Republic, the smaller prevalence was set to 40% of the prevalence of need in the Dominican adult population for each product domain. Similarly, for Sweden, the smaller prevalence was set to 30% of the prevalence of need in the Swedish adult population for each product domain. The reduced prevalences were based on the situation in other similar countries and differed because of the higher median age in Sweden compared to the Dominican Republic.

All analyses of needs for assistive products were performed using R Statistical Software (v4.3.3; R Core Team 2024). A linear mixed effects model was fitted to the rATA data from the 28 surveyed countries, using a power of the prevalence of need (*pN*) to make its distribution normal. The fixed effects by functional domain were the slopes for the median age (medAge) and for the Human Development Index (HDI) of the country (1). The random effects were the functional domains allowing for separate intercepts:

$$pN^{\frac{1}{4}}\;∼\;\mathrm{medAge}:\mathrm{domain}+\mathrm{HDI}:\mathrm{domain}+1|\mathrm{domain}$$

A preliminary regression identified two observations (out of 140) with outlying Cook's distance, indicating single data points with a large influence on the regression. These were removed before further analysis. The MCMCglmm function (Monte Carlo Markov Chain generalized linear mixed model) provided the regression coefficient with its 95% confidence intervals (CI) and an assessment of the quality of the MCMC simulation. The prediction function belonging to it was applied to all other 161 countries to estimate the prevalence of need and CI for each country and product domain. It was verified that no prevalence or CI boundary was negative due to a combination of low median age and high HDI. Prevalences and the boundaries of CIs were then raised to the fourth power and multiplied by the country's population to provide numbers of persons needing assistive products and CIs.

For the 28 rATA countries, CIs for the prevalence of need were calculated using the binomial distribution after rounding non-integer numbers of observations from the weighted survey data. These prevalence rates were then multiplied by the respective country's population to estimate the number of individuals requiring assistive products in each country. The results were subsequently aggregated to determine the global need for assistive products.

#### 2.3.3 Workforce required for providing assistive technology globally

The required sizes of the global workforce (*W_G_*) providing assistive technology were calculated using the equation:

$$W_{G}=W_{S}\frac{N_{G}}{N_{S}},$$

where *N_G_* represents the need for assistive products globally, *N_S_* represents the need for assistive products in Sweden, and *W_S_* represents the Swedish workforce providing assistive technology. The required workforce sizes were calculated for both the total workforce and the clinical and technical workforce in the five product domains.

To account for potential differences in workforce productivity across countries, a sensitivity analysis was performed to indicate how variations in productivity would impact the required global workforce. A deterministic approach was used, discussing parameters that may impact the size of the workforce (29, 30). Variations in productivity may be due to competence, such as having appropriate education, skills, and a mix of occupations (11). It may also depend on the effectiveness of the organization, for example, the possibility of task-sharing and coordination of services (11), or the distribution of the workforce when services are provided outside the clinic (7). Other factors that may influence productivity include production methods, materials, components, and infrastructure. As quantitative evidence on variations in workforce productivity is absent, a potential variation in productivity of 50% was deemed reasonable and therefore used in the sensitivity analysis for each product domain, using the following equations:

$$W_{G,\mathrm{low}}=0.5\;\times W_{S}\frac{N_{G}}{N_{S}}\;\mathrm{and}\;W_{G,\mathrm{high}}=1.5\;\times W_{S}\frac{N_{G}}{N_{S}}\;$$

All workforce calculations were performed using Microsoft 365 Excel, version 2412.

## 3 Results

### 3.1 Workforce in Sweden

The total workforce providing assistive technology across the three healthcare regions corresponded to 470 FTE, with 365 FTE in the clinical and non-clinical workforce and 105 FTE in the administrative workforce (see Table 1), which also presents the workforce categories by product domain. Specific occupations within each product domain are listed in the Supplementary Table. In the product domain of orthotics and prosthetics, it can be noted that the ratio between the clinical (orthotists, prosthetists, orthopedic shoemaker, seamstress, and associated orthotists and prosthetists) and the non-clinical (orthotic technicians, prosthetic technicians, and support staff) workforce is approximately 6–1 (see Supplementary Table).

**Table 1:** The workforce in the investigated regions in Sweden, by product domain.

Product domains	Total workforce (FTE^a^)	Clinical and non-clinical workforce (FTE)	Administrative workforce (FTE)
Cognition and communication	15.5	14.5	1.0
Hearing	105.3	82.5	22.8
Mobility and self-care	250.5	186.5	64.0
Orthotics and prosthetics	68.7	55.7	13.0
Vision	30.5	26.2	4.3^b^
All product domains	470.4	365.4	105.1

^a^ FTE, full-time equivalent.

^b^ In one region, hearing and vision services shared 6 administrative staff. They have been distributed proportionally to the total workforce for hearing and vision services in that region, i.e., 5 in hearing and 1 in vision.

In one of the studied healthcare regions, administration was shared between the vision and hearing centers. Consequently, the administrative workforce was allocated in proportion to the clinical and non-clinical workforce in these centers.

The extrapolated estimate of the total workforce across all product domains in Sweden is based on the workforce in the three healthcare regions (see Table 2). It corresponds to 5,298 FTE, i.e., 502 FTE per million population (FTEPM), of which the clinical and non-clinical workforce constitutes 4,115 FTE (390 FTEPM).

**Table 2:** Estimates of the workforce size and the number of persons needing assistive products in Sweden, by product domain.

Product domain	Total workforce (FTE^a^)	Total workforce (FTEPM^b^)	Clinical and non-clinical workforce (FTE)	Clinical and non-clinical workforce (FTEPM)	Number of people needing assistive products (millions) (95% CI)
Cognition and communication	174.6	16.5	163.3	15.5	0.40 (0.31; 0.51)
Hearing	1,185.3	112.3	929.1	88.1	0.49 (0.39; 0.61)
Mobility and self-care	2,821.2	267.4	2,100.4	199.1	0.49 (0.39; 0.61)
Orthotics and prosthetics	773.5	73.3	627.1	59.4	0.079 (0.044; 0.14)
Vision	343.2	32.5	294.7	27.9	0.050 (0.023; 0.099)
All product domains	5,297.8	502.1	4,114.6	389.9	

^a^ FTE, full-time equivalent.

^b^ FTEPM, full-time equivalent per million population.

In two of the three healthcare regions, 1,500 persons were listed as prescribers of assistive products in the product domains of cognition and communication, and mobility and self-care. Extrapolating the prescribers to all of Sweden based on the population ratio results in nearly 28,000 prescribers in these product domains.

### 3.2 Need for assistive products in Sweden

Table 2 also presents the estimated number of people in Sweden needing assistive products by product domain based on the rATA survey. They range from about 50,000 in the product domain of vision to about 500,000 in the product domain of hearing, and mobility and self-care.

### 3.3 Needs for assistive products globally

Table 3 presents the estimated number of people needing assistive products within the five product domains based on rATA surveys in 28 countries and regression modeling for 161 countries: mobility and self-care 396 (95% CI: 380–411) million; hearing 158 (150–167) million; cognition and communication 116 (109–122) million; orthotics and prosthetics 116 (108–123) million; and vision (excluding ordinary spectacles) 77 (72–81) million.

**Table 3:** Global estimates of the number of people needing assistive products and the size of the required workforce, by product domain.

Product domain	Number of people needing assistive products (millions)	Total workforce (million FTE^a^)	Total workforce (FTEPM^b^)	Clinical and non-clinical workforce (million FTE)	Clinical and non-clinical workforce (FTEPM)
Cognition and communication (95% CI)	115.5 (108.6; 122.4)	0.050 (0.037; 0.069)	6.2 (4.7; 8.5)	0.047 (0.035; 0.064)	5.8 (4.4; 7.9)
Hearing (95% CI)	158.3 (150.0; 166.6)	0.38 (0.29; 0.050)	47 (36; 62)	0.30 (0.23; 0.39)	37 (28; 49)
Mobility and self-care (95% CI)	395.5 (379.7; 411.4)	2.27 (1.77; 2.94)	281 (219; 364)	1.69 (1.23; 2.19)	209 (164; 271)
Orthotics and prosthetics (95% CI)	115.6 (108.4; 122.8)	1.13 (0.62; 2.15)	140 (76; 265)	0.92 (0.50; 1.74)	113 (62; 215)
Vision (95% CI)	76.5 (71.6; 81.4)	0.52 (0.24; 1.17)	65 (30; 145)	0.45 (0.21; 1.01)	55 (26; 125)
All product domains		4.36 (2.98; 6.83)	539 (368; 845)	3.40 (2.31; 5.40)	421 (285; 667)

^a^ FTE, full-time equivalent.

^b^ FTEPM, full-time equivalent per million population.

### 3.4 Workforce required for a high level of access to assistive technology globally

Table 3 also presents the estimated size of the required global workforce to provide assistive technology at a high level of access. The total workforce corresponds to 4.4 (95% CI: 3.0–6.8) million FTE [539 (368–845) FTEPM], including a clinical and non-clinical workforce of 3.4 (2.3–5.4) million FTE [421 (285–667) FTEPM] (Table 3). Within the five product domains, the estimated sizes of the required clinical and non-clinical workforces are: mobility and self-care 1.7 (1.3–2.2) million FTE; orthotics and prosthetics 0.9 (0.5–1.7) million FTE; vision 0.5 (0.2–1.0) million FTE; hearing 0.3 (0.2–0.4) million FTE; and cognition and communication 0.05 (0.04–0.06) million FTE.

Extrapolating the number of prescribers in Sweden to the global level, using the ratio between the sum of the needs for assistive products in the product domains of cognition and communication, and mobility and self-care, suggests that an estimated 16.0 (95% CI: 12.3–21.1) million prescribers would be required in these product domains.

### 3.5 Sensitivity analysis

Table 4 presents the results of the sensitivity analysis by product domain. It suggests that the point estimate of the required global total workforce providing assistive technology may fall within an interval between 2.2 and 6.5 million FTE, while the point estimate of the required global clinical and non-clinical workforce may fall within 1.7–5.1 million FTE.

**Table 4:** Sensitivity analysis of point estimates of the global total workforce and the global clinical and non-clinical workforce, by product domain.

Product domain	Total workforce, low (million FTE^a^)	Total workforce, high (million FTE)	Clinical and non-clinical workforce, low (million FTE)	Clinical and non-clinical workforce, high (million FTE)
Cognition and communication	0.025	0.076	0.024	0.071
Hearing	0.19	0.57	0.15	0.45
Mobility and self-care	1.14	3.41	0.85	2.54
Orthotics and prosthetics	0.57	1.70	0.46	1.38
Vision	0.26	0.78	0.22	0.67
All product domains	2.2	6.5	1.7	5.1

^a^ FTE, full-time equivalent.

## 4 Discussion

### 4.1 Workforce estimates

Aiming to estimate the global workforce required to provide assistive technology excluding ordinary spectacles at a high level of access, this modeling study suggests that a total workforce of 4.4 (95% CI: 3.0–6.8) million FTE would be required, of which the clinical and non-clinical workforce constitutes 3.4 (2.3–5.4) million FTE. In addition, about 16.0 (12.3–21.1) million prescribers of mobility, self-care, cognition, and communication products may be required, with assistive product prescription being part of their responsibilities.

The point estimates of the global workforce providing assistive technology correspond to a total workforce of 539 FTEPM and a clinical and non-clinical workforce of 421 FTEPM. These figures are slightly higher than in Sweden, which may be explained by larger proportions of people needing assistive products from labor-intensive product domains globally, such as orthotics and prosthetics, and mobility and self-care, and smaller proportions needing assistive products from less labor-intensive product domains, such as cognition and communication. This illustrates the importance of tailoring the composition of the workforce to the distribution of the assistive product needs of individual countries.

The findings showed that if the productivity levels fluctuate by up to 50% compared to the situation in Sweden, the global point estimate for the total workforce providing assistive technology could range from 2.2 and 6.5 million FTE, while the clinical and non-clinical workforce could vary between 1.7 and 5.1 million FTE. Besides differences in the workforce composition across product domains, several factors impact the required workforce size within each product domain. These factors include the number of people needing assistive products, the organization of the service systems and centers, including geographic distribution and whether services are centralized or decentralized, the type and range of assistive products provided and the extent to which they are prefabricated, the competence of the workforce, the production methods and models, and the financial context (16, 31).

In the product domain of orthotics and prosthetics in an average country, the WHO has suggested 5–10 clinicians (prosthetists, orthotists, and associates) per million people and 10–20 non-clinicians (technicians and support staff) per million people supporting the clinicians (16) to meet the needs. The number of clinicians is usually higher in high-income countries, at 15–20 clinicians per million people or more (16). This conforms well with the situation in Sweden, where the findings in this study showed that the combined clinical and non-clinical workforce was nearly 60 FTEPM, whereas previous research suggested that the situation in the United Kingdom with about 26 clinicians and non-clinicians per million people indicated a shortage according to the WHO recommendation (12). According to our findings, the ratio between clinical and non-clinical workforce in Sweden was about 6:1 instead of 1:2 for an average country or 1:1 for specialized services, as indicated by WHO. This may be possible due to the availability and use of prefabricated products and components, and central production (customized manufacturing and adaptations).

As mentioned above, the size of the workforce may depend on its competence. The global estimates for the clinical and non-clinical workforce in the five assistive product domains were based on the Swedish workforce, consisting of several occupations with different competencies. In Sweden, the clinical workforce was essentially health professionals and health-associated professionals (the majority with at least a bachelor's degree). Still, they included a few occupations with discontinued education programs (upper secondary school/high school), for example, orthopedic shoemakers. Swedish counselors within cognition, communication, mobility, or self-care require a background as a health professional or a health-associated professional. Technicians in Sweden may have had a more varied educational background, from apprentice and post-secondary education to engineering education, but also other technical areas, reflecting the broad range of required assistive technology professions suggested by DiGiovine et al. (32).

As a high-resource context with clearly defined specialist occupations within the clinical workforce in each specific assistive product area, Sweden may have an excess of specialists (11). Moreover, the impact of decentralization and task sharing on workforce requirements remains largely unexplored in this field. Consequently, the workforce density in Sweden may differ from the required workforce density in contexts with different resources or organizational approaches to assistive technology provision, which is important to consider when interpreting the findings of this study.

This study considered only the primary workforce involved in assistive technology provision at centers in Sweden operated by healthcare regions or by tender, along with the prescribers associated with them. These centers primarily contribute to the provision component of the WHO GATE 5P framework and, to some extent, the product component. Personnel engaged in the policy component—such as those responsible for funding, legislation, and regulations—as well as key aspects of the product component, including standards, design, and mass production of assistive products, were not included in this study. Moreover, the workforce providing assistive technology under legislations other than the Health and Medical Services Act was excluded. Therefore, the total global workforce needed across all components of the WHO GATE 5P framework is likely greater than estimated in this study.

To refine estimates of the global workforce required to provide assistive technology, further research is needed. Future studies should explore the workforce for each product domain separately, as they involve different occupations, job roles, and operations, and should incorporate data from countries with diverse systems for assistive technology provision.

### 4.2 Methodological considerations

Methodological choices were made to reduce the risk of overestimating the required global workforce. However, caution should be applied when interpreting the global estimates for planning and developing a global workforce for assistive technology provision.

Data on self-reported needs for assistive products were based on the same survey in both Sweden and other countries, increasing the validity of using the ratio between global needs and Swedish needs when estimating the size of the global workforce.

The representativeness of the three healthcare regions for Sweden as a whole in terms of access to assistive products can be assessed based on a report in which the Social Board of Health and Welfare has mapped variations and differences in access to assistive products across healthcare regions and municipalities in Sweden (33). They found that the conditions to get prescriptions and the regulations were similar, while varying fees affected access. However, the three healthcare regions in our study were relatively representative of Sweden regarding access.

The estimated total Swedish workforce to provide assistive technology (5,298 FTE) aligned well with but is slightly smaller than that of the Swedish Association of Local Authorities and Regions (5,470 FTE), indicating that the global workforce estimates may be conservative. This was further supported by the situation in some of the centers providing assistive technology in Sweden. One of the orthotic and prosthetic centers reported a shortage of three clinicians. Moreover, in the three healthcare regions, the orthotic and prosthetic centers used central production to varying degrees, and those working centrally may not all have been included, suggesting that the reported workforce figure was likely lower than the actual workforce size. However, the estimated workforce density for orthotists and prosthetists was consistent with data from the Swedish National Board of Health and Welfare, adding to the validity of the estimates. In one of the healthcare regions, the reconditioning of assistive products in the product domain of mobility and self-care was outsourced to an external actor whose staff was not included.

In Sweden, the roles of prescribers varied across the product domains, and the exact number of prescribers was difficult to compile due to a broad spectrum of occupations and multiple prescription systems. Together with the exclusion of the workforce providing assistive technology for education and employment, this has likely also contributed to the estimated workforce size being conservative.

The range of occupations across product domains was broad, and categorizing the workforce that provided assistive technology beyond clinical and non-clinical distinctions is complex. Some technicians may also work as clinicians, while health professionals, such as psychologists and social workers, may not work directly with the assistive products but with the individuals who need them. Furthermore, it has been noted that rehabilitation engineers, rehabilitation technologists, assistive technologists, and rehabilitation technicians often perform overlapping roles; for instance, an engineer may take on tasks typically assigned to a technologist or technician, and a technologist may perform duties usually handled by a technician (32). Hence, when applying the findings from this study in other contexts, the organizations of occupations involved in the provision of assistive technology must be adapted to local conditions.

## 5 Conclusion

Using assistive technology provision workforce data from a country with a high level of access to assistive products and needs data from 28 countries, this study suggests that a workforce of 4.4 million FTE (95% CI: 3.0–6.8 million) would be required to meet global assistive product needs. Given the conservative nature of this estimate, it provides a cautious basis for informing workforce development strategies worldwide. Establishing an international nomenclature for assistive technology occupations would support a systematic approach to competency development and staffing while enabling more effective international comparisons.

## Data Availability

The datasets presented in this article are not readily available. Swedish workforce data may be requested from the corresponding author. Data on assistive product needs may be requested from the Access to Assisitve Technology team, WHO at assistivetechnology@who.int.
