# Supplementary material for: Estimates of the global workforce required for providing assistive technology: a modeling study
Source: Front Rehabil Sci. 2025 Jul 8;6:1617624. doi: 10.3389/fresc.2025.1617624 (PMC12279701; doi:10.3389/fresc.2025.1617624)
Supplement: Supplementary file 1 [file Table1.docx]

Supplementary Material

# Supplementary Data

The workforce in the three healthcare regions and estimates of the Swedish workforce

| *Domains and workforce categories* | *Workforce in three healthcare regions (FTE*)* | *Estimated workforce in Sweden (FTE)* |
| --- | --- | --- |
| **Hearing** |  |  |
| Managers | 5 |  |
| Assistants | 10 |  |
| Secretaries | 4 |  |
| Administrators | 3.8 |  |
| Administrative workforce | 22.8 | 256.2 |
|  |  |  |
| Audiologists | 53 |  |
| Technicians or engineers | 14 |  |
| Hearing educators or special educators | 3 |  |
| Sign language interpreters | 5 |  |
| Social workers | 5 |  |
| Sign language teachers | 0.5 |  |
| Speech therapists | 1 |  |
| Assistant nurses | 1 |  |
| Clinical and non-clinical workforce | 82.5 | 929.1 |
|  |  |  |
| Total workforce - hearing | 105.3 | 1185.3 |
| **Vision** |  |  |
| Managers | 1.5 |  |
| Assistants | 1 |  |
| Administrators | 1.8 |  |
| Administrative workforce | 4.3 | 48.4 |
|  |  |  |
| Opticians | 5.2 |  |
| Occupational therapists | 7 |  |
| Social workers | 4.6 |  |
| Educators, IT or IT-staff | 2.9 |  |
| Educators, vision | 4.6 |  |
| Physiotherapists | 1 |  |
| Psychologists | 1 |  |
| Clinical and non-clinical workforce | 26.7 | 294.7 |
|  |  |  |
| Total workforce - vision | 30.5 | 343.2 |
| **Orthotics and prosthetics** |  |  |
| Managers | 2 |  |
| Administrators | 5 |  |
| Receptionists | 6 |  |
| Administrative workforce | 13 | 146.4 |
|  |  |  |
| Orthotists and prosthetists | 28.3 |  |
| Technician, Orthotic and Prosthetic | 7 |  |
| Orthopedic shoemakers | 15.4 |  |
| Seamstress | 2 |  |
| Associate prosthetists and orthotists | 2 |  |
| Support personnel | 1 |  |
| Clinical and non-clinical workforce | 55.7 | 627.1 |
|  |  |  |
| Total workforce - orthotics and prosthetics | 68.7 | 773.5 |
| **Mobility and self-care** |  |  |
| Managers | 18 |  |
| Assistants | 16 |  |
| Administrators | 20 |  |
| Purchasers | 4 |  |
| Secretaries | 6 |  |
| Administrative workforce | 64 | 720.8 |
|  |  |  |
| Counselors | 45.5 |  |
| Technicians | 85 |  |
| Engineers (assistive technology, medical, rehabilitation) | 12 |  |
| Repairer | 24 |  |
| Nurses | 3 |  |
| Counselor, incontinence | 1 |  |
| Speech therapists | 1 |  |
| Dieticians | 2 |  |
| Warehouse porters, managers (logistics), or drivers | 13 |  |
|  |  |  |
| Clinical and non-clinical workforce | 186.5 | 2100.4 |
|  |  |  |
| Total workforce - mobility and self-care | 250.5 | 2821.2 |
| **Cognition and communication** |  |  |
| Managers | 1 |  |
| Administrative workforce | 1 | 11.3 |
|  |  |  |
| Counselors | 8.5 |  |
| Technicians | 5 |  |
| Engineers | 1 |  |
| Clinical and non-workforce | 14,5 | 163.3 |
|  |  |  |
| Total workforce - cognition and communication | 15.5 | 174.6 |
| Total clinical and non-clinical workforce | 365.9 | 4114.6 |
| Total workforce | 470.4 | 5297.7 |

*FTE=full-time equivalent.
